# 3-D Modeling of Tomato Canopies Using a High-Resolution Portable Scanning Lidar for Extracting Structural Information

**DOI:** 10.3390/s110202166

**Published:** 2011-02-15

**Authors:** Fumiki Hosoi, Kazushige Nakabayashi, Kenji Omasa

**Affiliations:** 1 Graduate School of Agricultural and Life Sciences, The University of Tokyo, 1-1-1, Yayoi, Bunkyo-ku, Tokyo 113-8657, Japan; E-Mail: ahosoi@mail.ecc.u-tokyo.ac.jp; 2 Department of Agricultural Chemistry, Meiji University, 1-1-1, Higashi-mita, Tama-ku, Kawasaki-shi, Kanagawa 214-8571, Japan; E-Mail: nakakazu@isc.meiji.ac.jp

**Keywords:** canopy, crop, polygon, portable scanning lidar, 3-D, leaf area density, leaf area index

## Abstract

In the present study, an attempt was made to produce a precise 3D image of a tomato canopy using a portable high-resolution scanning lidar. The tomato canopy was scanned by the lidar from three positions surrounding it. Through the scanning, the point cloud data of the canopy were obtained and they were co-registered. Then, points corresponding to leaves were extracted and converted into polygon images. From the polygon images, leaf areas were accurately estimated with a mean absolute percent error of 4.6%. Vertical profile of leaf area density (LAD) and leaf area index (LAI) could be also estimated by summing up each leaf area derived from the polygon images. Leaf inclination angle could be also estimated from the 3-D polygon image. It was shown that leaf inclination angles had different values at each part of a leaf.

## 1. Introduction

The plant canopy plays an important functional role in cycling materials and energy through photosynthesis and transpiration, maintaining plant microclimates, and providing habitats for various species [1–4]. In crop canopies, the canopy structure has been investigated and related to characteristics such as light distribution within the canopy, light-use efficiency, yield, growth rate, and nitrogen allocation [5–8]. The canopy structure is often represented by leaf area density (LAD) in each horizontal layer, which is defined as one-sided leaf area per unit of horizontal layer volume [9]. The leaf area index (LAI) is then calculated by vertically integrating the LAD profile data. These indices can be utilized for crop management. However, both LAD and LAI are difficult to measure accurately without destructive sampling, so that it does not permit the measurement of intact crop structure as plants change over time with growth. In addition, LAD and LAI are spatial summaries of canopy structure and thus detailed structural information at each leaf level is not provided from those indices. If detailed canopy information at each leaf level could be easily extracted, the information would contribute greatly to good crop management, such as in yield estimation, optimizing fertilization and controlling crop water status. For this purpose, the shape of each leaf within canopy must be measured three-dimensionally. In previous studies, three-dimensional (3-D) digitization by ultrasonic or electromagnetic devices has been used to obtain structural information about the canopy at each leaf level [10,11]. Although this technique allows measurement of the detailed 3-D structure of plants at each leaf level through nondestructive means, this method is labor intensive because numerous components must be measured manually, point by point. Recently, a portable scanning lidar (light detection and ranging) instrument has been utilized to obtain 3-D structural properties of plants [12–21]. A portable scanning lidar can measure the distance between the sensor and a target based on the elapsed time between the emission and return of laser pulses (the time-of-flight method) or based on trigonometry (the optical-probe or light-section methods), so that 3-D information about the target can be obtained. The instrument can record many 3-D point cloud data of a target quickly and automatically and thus it eases the data collection of the canopy compared with above 3-D digitizing devices. This type of lidar has been used for estimating vertical LAD profile or LAI in broad-leaved canopies [15–17] and crop canopy [18]. However, these studies have been focused on estimations of LAD and LAI rather than structural information at each leaf scale. On the other hand, a high-resolution portable scanning lidar with the range resolution of about 1 mm has been used for capturing 3-D shape of each leaf of small potted plants [12,22]. Lidar’s ability of quick and automatic data collection worked well in those studies, so that the 3-D shape was easily captured. Although only small potted plants were treated in those studies, it is significant that the high-resolution portable scanning lidar allowed easy structural measurement of plants at each leaf scale. By enhancing the technique, structure of crops with larger canopy may be able to be measured at each leaf level. Therefore, in the present study, a crop with larger canopy is measured by a high-resolution portable scanning lidar. Based on the obtained data, the method to extract structural information of each leaf has been demonstrated.

## 2. Experimental Section

**Figure 1.**
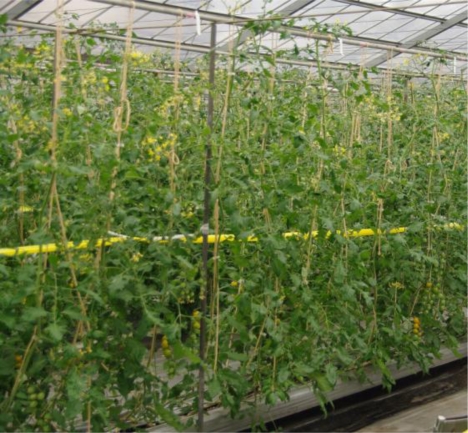
Photograph of tomato (*Lycopersicon esculentum* Mill.) canopy.

The experiment was conducted on 20 November 2009 using tomato (*Lycopersicon esculentum* Mill.) plants, which were cultivated in a greenhouse using coral sand as the culture medium (Figure 1). The height of the canopy was about 1.8 m at the measurement date.

**Figure 2.**
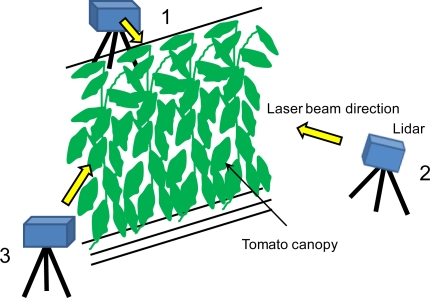
A schematic view of the tomato canopy measurement by the portable scanning lidar. Arrows show the directions corresponding to the center of laser beam scan from each of the measurement positions of 1 to 3.

A portable high-resolution scanning lidar that calculates distances based on trigonometry (a modified TDS-130L 3-D laser scanner; Pulstec Industrial Co., Ltd., Japan) was used to measure the tomato canopy structure. The range and scan resolutions are about 1 and 2 mm, respectively, at a measurement range of about 5 m. A rotating mount with a stepper motor and a galvano mirror within the lidar head automated the horizontal and vertical scanning. Figure 2 illustrates a schematic view of the tomato canopy measurement by the portable scanning lidar with three scanning positions (1 to 3) surrounding the canopy. Arrows in Figure 2 show the directions of the laser beam scan from each of the measurement positions of 1 to 3. In position 1, the azimuth laser beam direction was perpendicular to the direction of the row of the tomato canopy. Positions 1 to 3 were 120° apart from each other in terms of azimuth direction. Distances between the tomato canopy and lidar positions were about 5.0 m. The zenith angle of laser beams was 94 ± 13° at all lidar positions. It took about 15 minutes for a measurement from each position. Leaf shapes cannot be captured accurately if the leaves move due to air movement. Thus, lidar measurements must be conducted under conditions without any influence of air movement. The data taken from three positions had individual coordinate systems. The data were registered using the iterative closest-point (ICP) algorithm [23], so that three data sets had a common 3-D coordinate system. The algorithm of the ICP starts with an initial estimate of corresponding points between two lidar data sets measured from different positions. Based on the corresponding points, the data are co-registered through a rigid-body transformation. The transform was then iteratively refined by alternately choosing corresponding points in the lidar data and finding the best translation and rotation matrices that minimize an error metric based on the distance between them. This procedure was used for all pairs of lidar data. A certain region (about 0.34 m^3^), which was fully illuminated by enough laser beams, was selected from the point cloud data. Since each leaf or stem shape was distinguishable due to the precise image obtained by high-resolution portable lidar, the data corresponding to all the leaves within the region could be picked out manually one by one. Thereafter, the point cloud data of the leaves were converted into polygon images, where irregular triangle meshes (*i.e.*, polygons) were determined uniquely by arrangement of each point. Through this process, leaves were converted from point cloud images into polygon surface ones. Such surface images of leaves allowed to calculating the leaf area, so 30 leaves were chosen randomly from the polygon images and the areas were calculated. Actual areas of the corresponding 30 leaves were also measured using a commercially available desk top scanner (FB636U, Canon, Inc., Japan), where leaves were scanned as JPEG images and the areas were determined by multiplying the number of pixels the area per pixel [17]. Lidar-derived and actual leaf areas of each leaf were compared each other and the mean absolute percent error (MAPE) of lidar-derived leaf area was obtained. Areas of all leaves besides above 30 leaves within the selected region were also calculated. From the estimated leaf areas, spatial summaries of canopy structure, *i.e.*, LAD, LAI, were also estimated. The estimated leaf areas were summed up in each horizontal layer with the thickness of 20 cm, then the LAD in each layer was estimated. LAI was obtained by vertically integrating the LAD values. A leaf inclination angle is defined as the zenith angle of a normal of a point on a leaf surface. A polygon leaf surface image can provide normals of each point on the surface. Thus, leaf inclination angles of each point on a leaf can be derived from zenith angles of the normals of the polygon image. Based on this theory, a image that shows distribution of leaf inclination angle on a leaf surface was produced. In addition, five leaves were chosen from produced polygon leaf images and 20 points on a leaf surface were selected randomly in each leaf. Leaf inclination angles of selected points were obtained as zenith angles of normals of the points and then the mean and standard deviation values were estimated.

## 3. Results and Discussion

**Figure 3.**
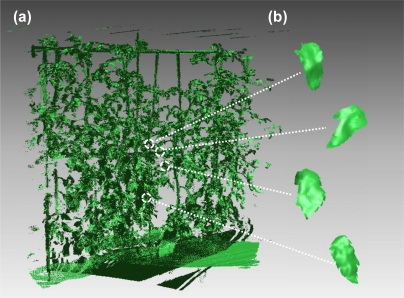
**(a)** A 3D point cloud image of tomato canopy obtained by a high-resolution portable scanning lidar and **(b)** example of polygon leaf images made from the lidar-derived point cloud data.

**Figure 4.**
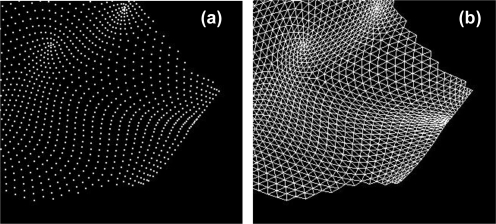
Close-up views of **(a)** a point cloud image of a certain leaf and **(b)** its conversion into the corresponding polygon image.

**Figure 5.**
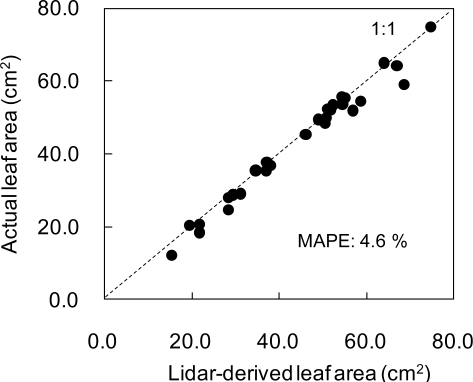
Comparison between leaf areas estimated from lidar-derived polygon leaf images and actual leaf areas. MAPE: Mean Absolute Percent Error.

The tomato canopy was scanned by laser beams from three positions and a point cloud image was obtained after registration, as shown in Figure 3(a). In the image, shading effect was added by changing the brightness of each point. Each leaf or stem shape is distinguishable due to the precise image obtained by high-resolution portable lidar. Examples of leaf polygon images made from the lidar-derived point cloud data are shown in Figure 3(b). It is observed in this figure that complicated curves and unevenness on each leaf surface are fully reproduced at the polygon images. The number of polygons per leaf ranged from 160 to 71,000, depending on leaf size. Figure 4(a,b) show close-up views of a point cloud image of a certain leaf and its conversion into the corresponding polygon image, respectively. It can be observed that the polygon image consists of irregular triangle meshes. From the polygon images, areas of leaves were calculated and compared with the actual ones, as shown in Figure 5. Although the areas derived from polygon images are a little underestimated, the lidar-derived leaf areas were very accurate with the MAPE of 4.6%.

**Figure 6.**
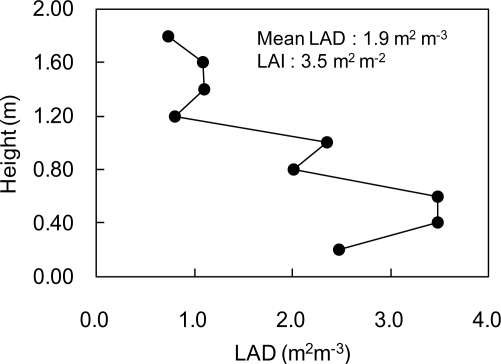
Vertical LAD profile of the tomato canopy estimated from lidar-derived leaf areas.

**Figure 7.**
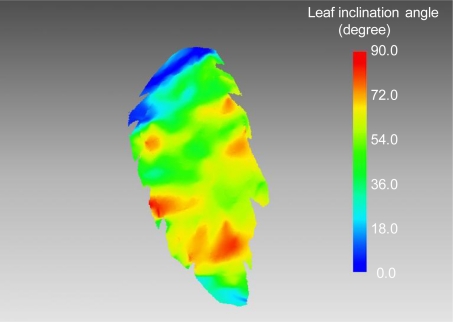
Distribution of leaf inclination angle on a polygon surface image of a certain leaf.

**Figure 8.**
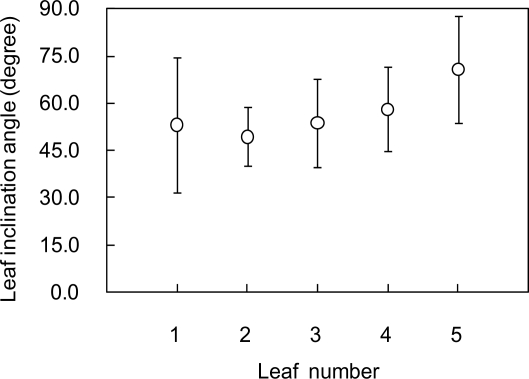
Leaf inclination angles estimated from polygon images of five leaves. White circles and error bars are mean and standard deviation values of each leaf, respectively.

Figure 6 shows the vertical LAD profile of the canopy estimated from lidar-derived leaf areas. LAD values of lower layers were 2.5–3.5 m^2^ m^−3^, while the ones of upper layers were around 1.0 m^2^ m^−3^. This shows that more leaves are distributed in lower layers. From the result, mean LAD and LAI were estimated as 1.9 m^2^ m^−3^ and 3.5 m^2^ m^−2^, respectively. Figure 7 shows the distribution of leaf inclination angle on a polygon image of a certain leaf. Leaf inclination angles were around 0° or 90° at some parts of the leaf, while the angles ranged from 36° to 72° for most of the leaf. It was confirmed that leaf inclination angle has a different value at each part of a leaf. That is also observed in a result of lidar-derived leaf inclination angles for five leaves shown in Figure 8. As seen in this result, the angles widely ranged in each of the leaves. In terms of accuracy of the estimated angles, our previous study [24] can be referred, in which a mean error of lidar-derived leaf inclination angles of thin leaves (wheat leaves) was 4.3° at 60 samples.

In previous studies [12,22], it has been demonstrated that the high resolution portable scanning lidar used in this study is able to precisely reproduce small potted plants at each leaf level. Also in this study, the lidar could depict a tomato canopy with a complicated structure so precisely that each leaf and stem shape were clearly distinguishable. This is due not only to the lidar’s resolution and accuracy, but also the several lidar positions surrounding the canopy.

If the lidar measurement is conducted at only one position, laser beams cannot fully illuminate all leaves, so that many leaves are not reproduced or incompletely reproduced. Thus, it is important to take several lidar positions for good reproduction of a crop canopy. On the other hand, an excessive number of lidar positions leads to longer measurement times. Thus, the number of the positions should be determined on consideration of laser beam coverage and measurement time. The present individual leaf level measurement could offer more useful information for crop management or diagnosis of plant status. For instance, it is usually difficult to diagnose degree of water stress nondestructively, but the present method would allow to diagnosing the water stress by quantifying the degree of leaf droop through the estimates of each leaf area and inclination angle. With respect to time needed for lidar measurements from several positions, it took only several ten minutes. In spite of such short time measurement, variety of canopy information, *i.e.*, leaf shape, leaf area, leaf inclination angle, LAD, LAI, *etc.* could be extracted. This means that this method is able to offer a variety of canopy information even at repeated measurements in a short period of time. This advantage is beneficial, especially for structural measurement of crops over time because crop canopy structure typically changes in a short period of time. In this study, the processing after lidar measurements took a few hours because some manual operations were included in the process. For more efficient modeling, more automated processes after the measurements should be studied in the future works. Although only structural information of canopy is treated in this study, it has been demonstrated in previous studies [12,22] that 2-D images that give physiological information, e.g., a thermal image, reflectance image, chlorophyll fluorescence image, can be mapped on the lidar-derived 3-D polygon image using a texture-mapping technique [25]. This allows physiological information about a plant to be considered together with the structural information. Although this was tried only for small potted plants in the previous studies, a similar technique could be applied to larger canopies as in the present study because each leaf within the canopy is converted into a polygon image. In future work, this combination technique should be applied to crop canopies to extract useful information about crop status and facilitate crop management.

## 4. Conclusions

In the present study, an attempt was made to produce a precise 3D model of a crop (tomato) canopy using a portable high-resolution scanning lidar to extract nondestructively detailed structural information of crop canopy at each leaf level. For this purpose, the tomato canopy was scanned by the lidar instrument from three positions surrounding the canopy. Through the scanning, point cloud data of the canopy were obtained and they were co-registered. Then, points corresponding to leaves were extracted and converted into polygon leaf images. From the polygon images, leaf areas were accurately estimated. Moreover, it was proven that other detailed canopy information, *i.e.*, LAD, LAI, leaf inclination angle, could be estimated from the produced 3-D canopy image. As a future step, the demonstrated 3-D crop canopy image and other 2-D images that include physiological information of the crop canopy should be combined and then analyzed for better understanding of crop status.
